# A Rare Case of Primary Hyperaldosteronism Resulting in Severe Hypokalemia

**DOI:** 10.7759/cureus.82069

**Published:** 2025-04-11

**Authors:** Saad Sameer, Aya J Qassim, Ali Aqel, Yousef H Al-Asa'd, Shaefiq Babu Thappy

**Affiliations:** 1 Internal Medicine, Hamad General Hospital, Doha, QAT; 2 Nephrology, Hamad General Hospital, Doha, QAT

**Keywords:** adrenal adenoma, endocrinology, hypokalemia related medical emergencies, multi-disciplinary care, nephrology, primary hyperaldosteronism, robotic adrenalectomy

## Abstract

Hyperaldosteronism often clinically manifests as hypertension with mild to moderate hypokalemia. However, in rare cases, it can also present primarily with severe hypokalemia. In this case report, we will be discussing our patient, a 43-year-old woman who presented with abdominal cramps and vomiting, along with severe muscle cramps resulting in an inability to walk. She was found to have an undetectable level of serum potassium and prolonged QT interval on her ECG. After aggressive electrolyte correction and further imaging and endocrinology studies, she was diagnosed to have primary hyperaldosteronism. She was appropriately referred for urgent outpatient adrenalectomy, which turned out to be curative. Our case highlights the importance of timely diagnosis, interventions, and multidisciplinary care for patients with primary hyperaldosteronism.

## Introduction

Hyperaldosteronism is a condition marked by excessive secretion of aldosterone. It presents a significant challenge in clinical practice due to its diverse etiologies and potential for severe complications. It can be classified into either primary or secondary hyperaldosteronism. Primary hyperaldosteronism occurs when there is an excess of mineralocorticoids independent of the renin-angiotensin system. This excess hormone secretion typically stems from tumors or abnormal growth in the glomerular zone of the adrenal glands. Clinically, hyperaldosteronism often manifests as hypertension and electrolyte imbalances, particularly hypokalemia [1]. The condition was first described in 1955 by Jerome W. Conn, who reported a young woman with a seven-year history of weakness and periodic paralysis of her lower limbs [2].

Here, we present a case report of one of our patients who presented to our hospital with a one-week history of similar muscle cramping and periodic paralysis of her lower limbs. She had undetectably low levels of potassium, which is rare for this etiology, and was subsequently found to have primary hyperaldosteronism.

## Case presentation

Our patient is a 43-year-old Indonesian woman with a history of hypertension and dyslipidemia who presented to the Hamad General Hospital Emergency Department (ED) in Doha, Qatar, with a one-week history of cramping abdominal pain with vomiting, followed by cramps in both legs along with an inability to walk on the last day before presentation. Her home medications were amlodipine 5 mg and fenofibrate 200 mg daily, and she was following up with a primary care physician for these. She works as a housemaid and has never had such symptoms before. Her only surgical history was that of a lower segment C-section a few years ago, which was unremarkable.

Upon eliciting further history, she reported that she had been having upper abdominal pain with a few episodes of occasional non-bilious, non-bloody vomiting for one week, with no predisposing risk factors. In the last 24 hours before the presentation, she started having leg cramps and weakness, which worsened to a point where she couldn’t walk. Upon a systematic review of systems, she also reported intermittent palpitations with chest discomfort over the last three months and frequent micturition without burning.

In the ED, she was hypertensive with readings of 160/96 and a heart rate of 58 bpm. She was 1.58 m tall and weighed 47 kg, thereby having a BMI of 18.8 kg/m^2^. On examination, she was completely awake, alert, and responding to questions. Her neurological examination was remarkable for 2/5 power in both lower limbs, while the upper limbs had intact power. Deep tendon reflexes were normal in the knees, and sensation was preserved throughout. Pupils were 3 mm bilaterally and reactive, and the rest of the neurological examination was normal with no focal deficits.

**Figure 1:**
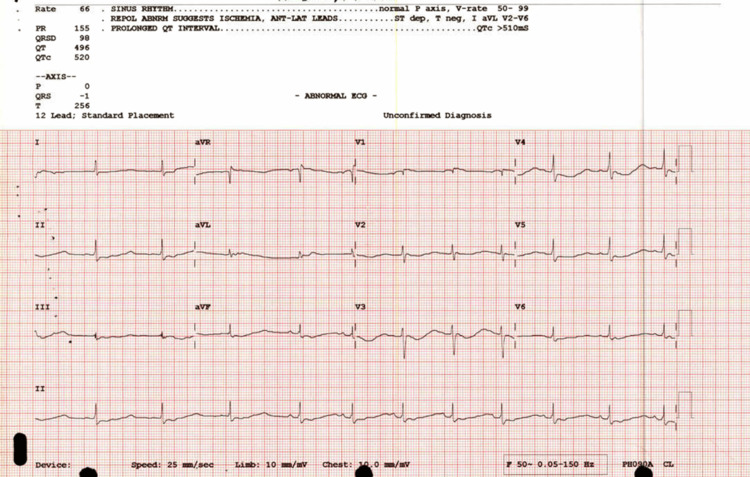
First ECG; prolonged QT interval of 520 ms.

An ECG was taken, which showed a prolonged QT interval of 520 ms with U waves (Figure 1).

**Figure 2:**
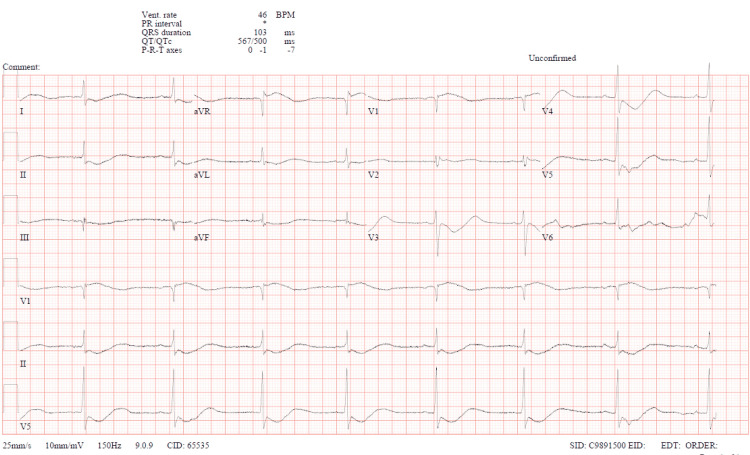
Subsequent 12-lead ECG strip showing QTc of 500 ms. QTc: corrected QT interval.

The initial two readings of potassium in the blood were unrecordable, and she was immediately started on IV and oral potassium replacement and admitted to the ICU for further management. Figure 2 shows the subsequent ECG strip taken after potassium correction was started.

After receiving 40 mEq potassium chloride (KCl) via IV, her K level was 1.5 mmol/L (the minimum recordable K in our lab is 1.5 mmol/L). Surprisingly, the only symptoms she was still feeling were the cramps in the lower limbs bilaterally. Other electrolytes and lab tests are shown in Table 1.

**Table 1:** Results of serum and urine electrolytes and ensuing endocrine investigations. ACTH: adrenocorticotropic hormone.

Parameter	Result	Normal range
Potassium (K)	1.5 mmol/L	3.5 – 5.3 mmol/L
Sodium (Na)	152 mmol/L	133 – 146 mmol/L
Phosphorus (PO₄)	0.46 mmol/L	0.8 – 1.5 mmol/L
Bicarbonate (HCO₃)	34 mmol/L	22 – 29 mmol/L
AST (aspartate aminotransferase)	110 U/L	8 – 32 U/L
ALT (alanine aminotransferase)	132 U/L	7 – 33 U/L
Parathyroid hormone (PTH)	69 pg/mL	15 – 65 pg/mL
Aldosterone (pmol/L)	3410 pmol/L	49 – 644 pmol/L
Plasma renin activity (PRA)	<0.14 ng/mL/hr	0.29– 6.12 ng/mL/hr
Aldosterone:renin ratio (ARR)	>877	≤ 20
AM cortisol (nmol/L)	33.5 nmol/L	138 – 689 nmol/L
PM ACTH (pg/mL)	15.9 pg/mL	7.2 – 63.3 pg/mL
24-hour urine analyte	Result (units)	Normal range
U24 calcium	12.8 mmol/24 hrs	2.5 - 7.5
U24 chloride	784 mmol/24 hrs	110 - 250
U24 creatinine	6.16 mmol/24 hrs	7.00 - 14.00
U24 phosphorus	46 mmol/24 hrs	15 - 50
U24 potassium	196 mmol/24 hrs	25 - 125
U24 sodium	725 mmol/24 hrs	40 - 220

She was then kept on an aggressive IV and oral potassium correction protocol, and her potassium levels normalized within 36 hours. She was stepped out of the ICU and transferred to the medical floor for further workup.

In the ward, the rest of her endocrine workup revealed a parathyroid hormone (PTH) level of 69 pg/mL, an aldosterone level of 3410 pmol/L, a plasma renin activity level of <0.14 ng/mL/hr, and an aldosterone:renin ratio of >877 (Table 1). She also had a normal AM cortisol level of 33.5 nmol/L and PM adrenocorticotropic hormone (ACTH) of 15.9 pg/mL. She was still receiving oral potassium supplementation in the ward.

**Figure 3:**
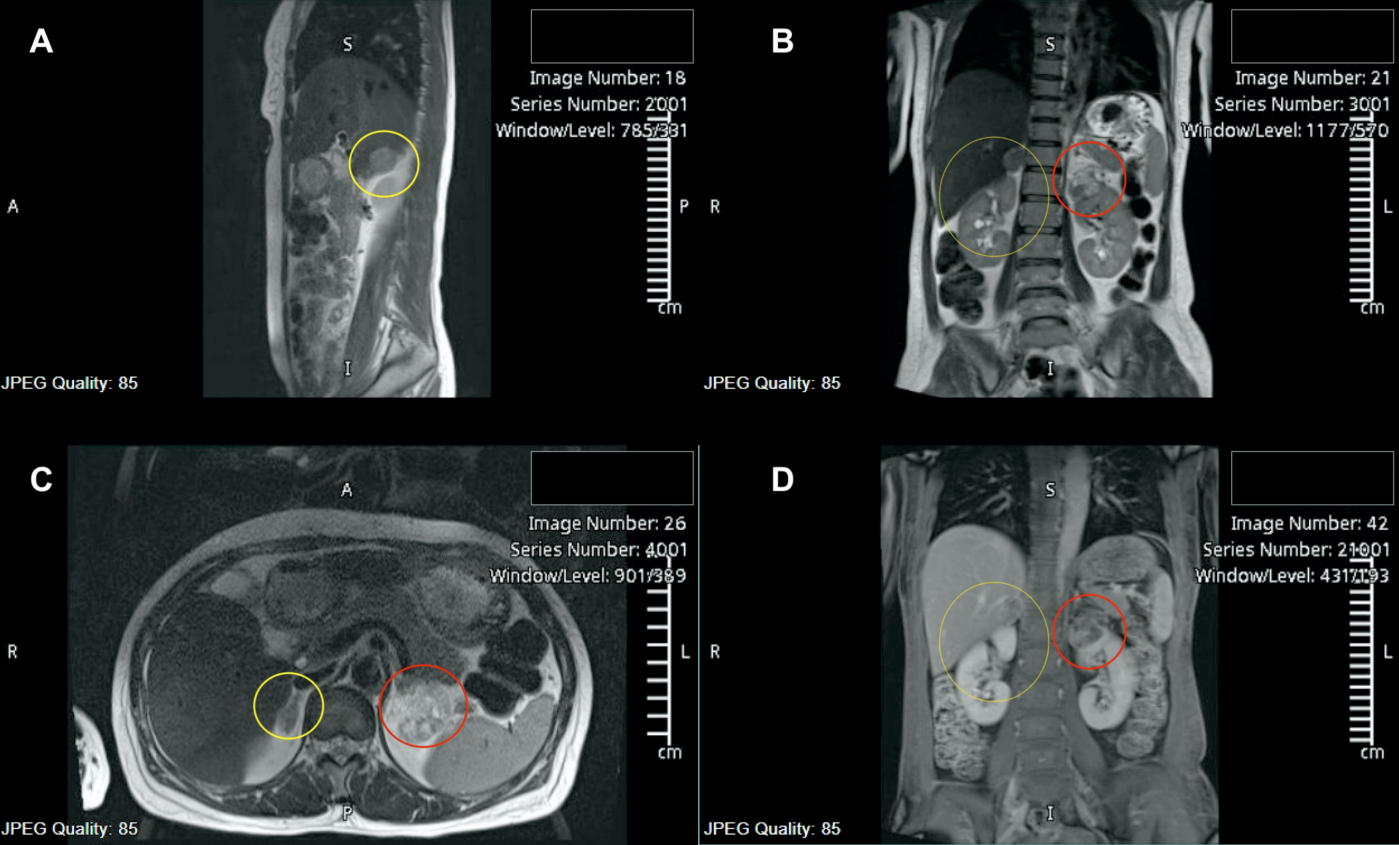
MRI abdomen depicting the right sided adrenal adenoma (circled in yellow), sized 2.5 x 2 cm in different planes. The lesion on the upper pole of the left kidney, sized 3.6 x 3.4 cm (circled in red) was later investigated and turned out to be an angiomyolipoma. (A) Sagittal view, T2 weighted; (B) coronal view, T2 weighted; (C) axial view, T2 weighted; (D) coronal view, T1 weighted.

She was presumptively diagnosed with primary versus secondary hyperaldosteronism, and further workup was then done, including an MRI of the adrenal glands, which revealed a well-defined lesion in the right adrenal gland measuring 2 cm in maximum dimension showing significant loss of signal intensity on T1 opposite phase (cortisol to serum insulin {CSI} ratio less than 0.71), suggestive of a lipid-rich adrenal adenoma (Figure 3). A few simple cysts were also visualized in the liver and spleen. There was a suspicious-looking lesion in the upper pole of the left kidney, sized 3.6 x 3.4 cm, which was later worked up for renal cell carcinoma (RCC) and pheochromocytoma and turned out to be an angiomyolipoma. The left adrenal gland was unremarkable on the MRI.

A bilateral renal artery Doppler was also done to rule out renal artery stenosis, and it was unremarkable.

Bilateral adrenal vein sampling (AVS) was then done as the patient was over 35 years of age and agreed to possible surgery, and the results are depicted in Table 2.

**Table 2:** Results from the adrenal venous sampling (AVS). The high aldosterone-to-cortisol and lateralization ratio confirm the lateralization of the hyperaldosteronism to the right side. IVC: inferior vena cava.

Parameter	Normal values	Patient's AVS results
Left adrenal to IVC cortisol (ng/dL)	3.0 – 10.0 ng/dL	3.8 ng/dL
Right adrenal to IVC cortisol (ng/dL)	3.0 – 10.0 ng/dL	9.2 ng/dL
Interpretation of cortisol values	Cortisol gradient should be >3 times higher in adrenal vein than peripheral blood	Confirmed correct catheterization due to appropriate cortisol gradient (9.2/3.8 ≈ 2.42)
Left adrenal to IVC aldosterone (cortisol corrected, ng/dL)	4–12 ng/dL	3.5 ng/dL
Right adrenal to IVC aldosterone (cortisol corrected, ng/dL)	4–12 ng/dL	21.9 ng/dL
Aldosterone-to-cortisol ratio (A/C) lateralization	Normal if < 4 (suggests no lateralization)	6.2:1

The patient’s hypertension while inpatient was managed with lisinopril, titrated up to 10 mg daily, and she was also started on spironolactone shortly after admission, which was later titrated up to 100 mg daily.

Urology, endocrinology, and nephrology teams were involved in the care, and the plan was made for a right adrenalectomy by the urology team. She underwent a successful robotic right adrenalectomy four weeks after her initial presentation and was shortly after discharged with appropriate follow-up.

The results from the biopsy of the resected lesion revealed an adrenal cortical adenoma, 2.5 cm with a Modified Weiss score of 0/7 (clear cells, no necrosis, 0/50 HPF mitotic range, absent atypical mitoses, no capsular invasion).

Upon her three-month follow-up, her blood tests revealed normal electrolytes, and all her symptoms had disappeared. She was continued on spironolactone 50 mg and lisinopril 10 mg daily, with controlled blood pressure readings after the surgery.

## Discussion

Primary hyperaldosteronism (PA) is the most common endocrine cause of secondary hypertension, exhibiting a varying prevalence in the literature that can reach up to 11% among patients with hypertension [3]. Approximately two-thirds of cases are caused by unilateral adrenal adenoma, while one-third are caused by bilateral adrenal hyperplasia, and less than 1% of cases are caused by adrenocortical carcinoma [4]. Most cases of PA present with normokalemia, with hypokalemia detected in only 9-37% of these patients [1]. Two factors contribute to hypokalemia in patients with primary hyperaldosteronism: high levels of aldosterone, which enhance potassium secretion through the kidneys, and an appropriate amount of sodium and water in the distal part of the nephron [5]. Hypokalemia in PA patients often maintains stable potassium levels; this is because the potassium-retaining effect of hypokalemia itself counterbalances the effect of high aldosterone levels. Severe hypokalemia in the setting of PA usually happens in patients with an added contributing factor of hypokalemia.

A case published by Goto et al. in 2009 highlighted severe hypokalemia-induced rhabdomyolysis in a patient with PA; the patient had taken two weeks of thiazide diuretics prior to admission [6]. In our case, the patient was found to have a severe, initially unrecordable serum potassium level. Although the patient experienced a few episodes of vomiting, which may have influenced the hypokalemia, it is unlikely to have caused it to drop so drastically. Additionally, no other contributing factors for this severe hypokalemia were identified.

Hypokalemia can manifest as cardiac arrhythmia, muscle weakness, myopathy, or even rhabdomyolysis and acute kidney injury in rare cases. A case reported by Hsieh et al. in 2011 revealed severe hypokalemia-induced ventricular fibrillation in patients with PA [7]. In our case, the patient complained of intermittent palpitations starting three months before presentation, but admission ECG revealed no serious arrhythmias and showed a prolonged QT interval with U waves. Another case report by Andrawus et al. in 2018 reported a woman walking into the emergency department with isolated lower limb weakness who was also found to have a unilateral adrenal adenoma consistent with Conn's disease [8].

There are only a few cases reported in the literature of hypokalemic myopathy or rhabdomyolysis as an initial presentation of PA [9-15]. Clinicians should consider the possibility of PA in patients presenting with muscle weakness in the setting of hypokalemia. Although presentations can sometimes be confounding, Tang et al. in 2011 reported ten patients who presented with a picture of polymyositis, which has a completely different pathophysiology and treatment options [16]. 

Patients with PA exhibit increased cardiovascular disease prevalence compared to patients with primary hypertension, and the increased risk is mainly due to the activation of cardiac mineralocorticoid receptors by increased aldosterone secretion [3]; this is independent of high blood pressure. This increased cardiovascular risk includes increased risk of myocardial infarction, heart failure, atrial fibrillation, and stroke, as have been reported in the literature [17,18]. Due to the risk of these severe complications and considering that PA can be a surgically curable disease, early diagnosis and treatment are important. The main treatment for unilateral PA is laparoscopic adrenalectomy. On the other hand, the bilateral disease is treated with mineralocorticoid receptor antagonists, i.e., spironolactone or eplerenone. Patients with unilateral adenoma often exhibit favorable responses after surgical excision in terms of normalization of BP, resolution of muscle weakness, and hypokalemia [9,10].

Moreover, mineralocorticoid receptor antagonists such as spironolactone decrease mortality in patients with heart failure in the setting of elevated aldosterone [17]. Pemayun et al. in 2017 reported a case of a patient with biventricular cardiac hypertrophy that in part is attributed to PA; after surgical removal of the adrenal adenoma, the patient’s left ventricular mass and index ventricular geometries were improved [19]. Our patient was admitted to the medical ICU, received potassium replacement, and was started on spironolactone, which was titrated up to 100 mg. Workup showed right adrenal adenoma with positive lateralization of hyperaldosteronism to the right side. Four weeks after starting spironolactone, the patient underwent a robotic right adrenalectomy. Follow-up confirmed resolution of symptoms and normalization of potassium.

Our patient had an unusual presentation of PA, with severe hypokalemia (initially undetectable potassium serum level), in which proper management led to the normalization of potassium and the resolution of symptoms.

## Conclusions

Our case report sheds light on a rare potential clinical presentation, diagnostic approach, and management strategies for primary hyperaldosteronism. Given the potential for severe complications and the fact that primary aldosteronism (PA) can be surgically treatable, timely diagnosis and intervention are crucial.
